# Supplementary figures and images for: Characterisation of protein isoforms encoded by the Drosophila Glycogen Synthase Kinase 3 gene shaggy
Source: PLoS One. 2020 Aug 6;15(8):e0236679. doi: 10.1371/journal.pone.0236679 (PMC7410302; doi:10.1371/journal.pone.0236679)

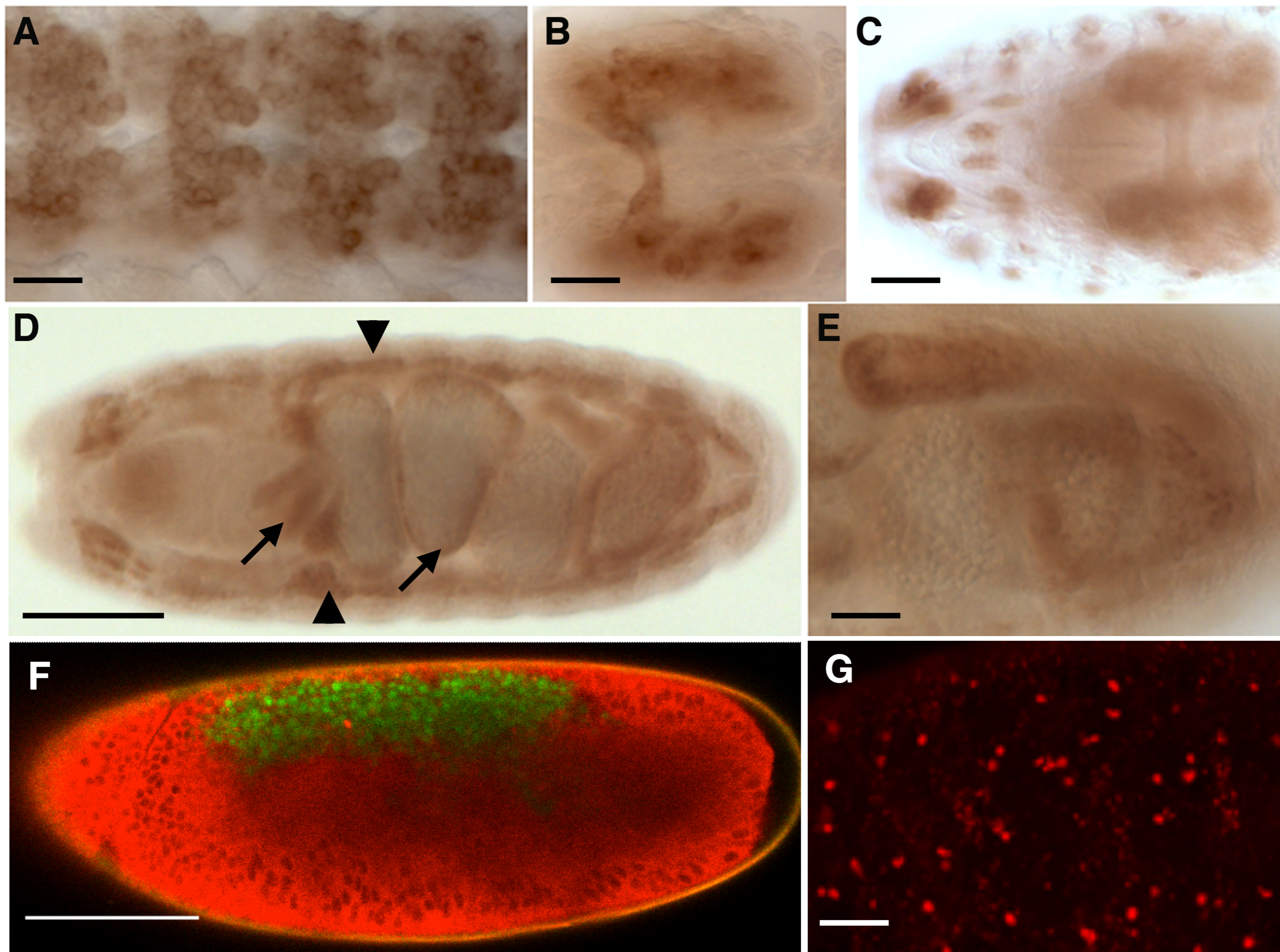

**Figure S1**

Supplement: S1 Fig — (A-C) anti-FLAG immunohistochemistry of Sgg-PA expression in the ventral neuroectoderm of a stage 10 embryo (A), the brain from a stage 16 embryo (B) and the head region showing various sensory organs (C). Scale = 20μm. (D and E) dorsal view of a stage 16 Sgg-PB embryo showing expression in the gut (arrows) and mesoderm (arrowheads), Scale = 100μm, and lateral view of hindgut (E) Scale = 20μm. (F) blastoderm embryo expressing mCherry tagged Sgg-PB and YFP tagged Sgg-PA, demonstrating ubiquitous Sgg-PB, the YFP signal represents yolk cell autofluorescence, Scale = 100μm. (G) close up of punctate epidermal expression in a Sgg-PB mCherry stage 16 embryo, Scale = 20μm. (PDF) [file pone.0236679.s001.pdf]

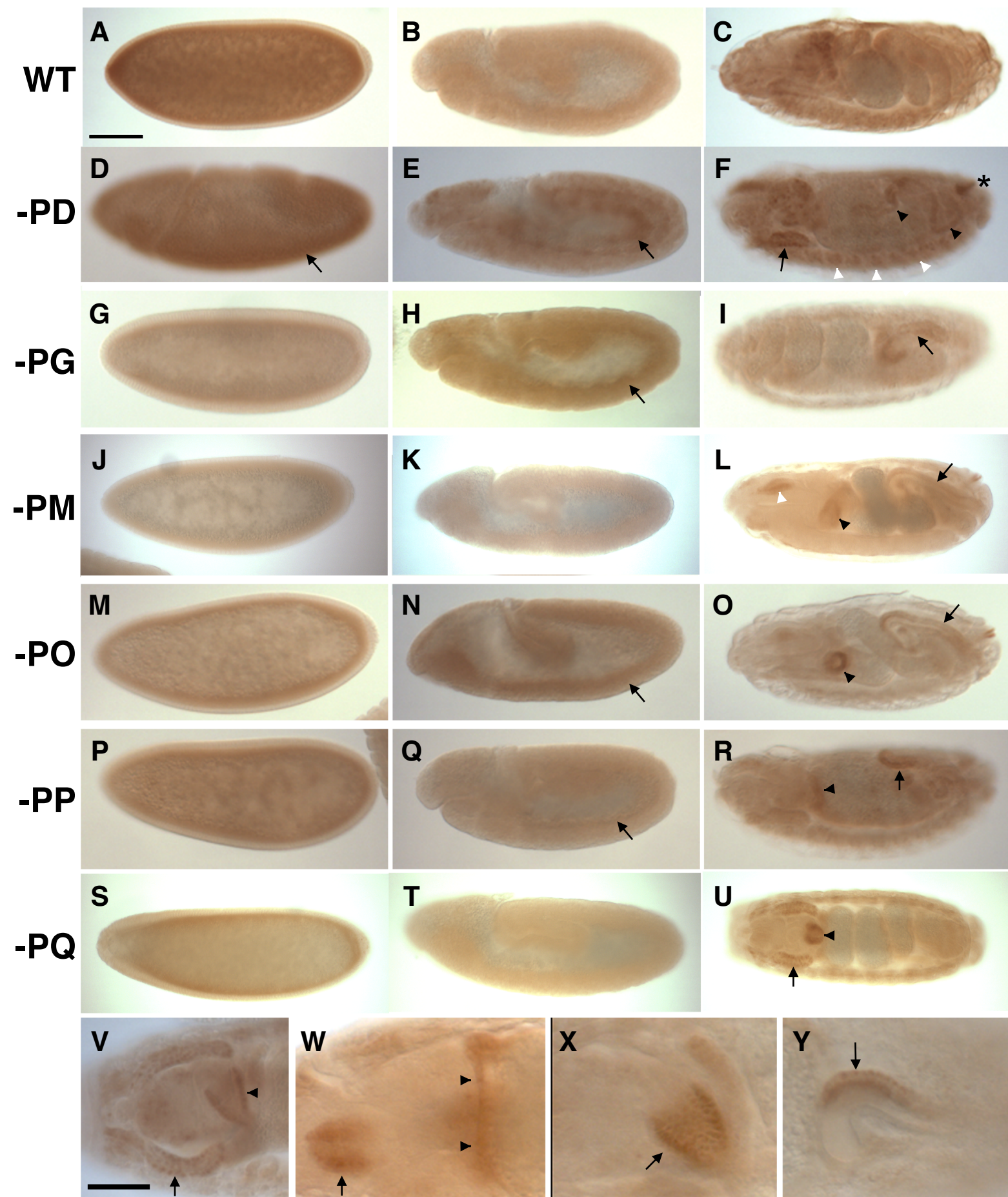

**Figure S2**

Supplement: S2 Fig — A-U) Anti-flag staining of embryos with the indicated tagged proteoforms at stage 5–6 (blastoderm, left column), 10–11 (germband extension, middle column) and 16 (late embryogenesis, right column). All embryos oriented anterior to the left dorsal to the top except U, which is a dorsal view. See text for full details of expression. (D and E), arrows = meseoderm; (F) arrow = salivary gland, arrowheads = malphigian tubules, asterisk = posterior spiracles; (H) arrow = mesoderm; (I) arrow = hindgut; (L) arrow = hindgut, arrowhead = foregut, white arrowhead = pharynx; (N) arrow = mesoderm; (O) arrow = hindgut, arrowhead = proventriculus; (Q) arrow = mesoderm; (R) arrow = hindgut, arrowhead = anterior midgut; (U) arrow = salivary gland, arrowhead = proventriculus. Scale bar in A = 100μm. (V-Y) Close up dorsal views highlighting: (V) Sgg-PD expression in the salivary gland (arrow) and proventriculus (arrowhead); (W) Sgg-PG in the foregut (arrowhead) and anterior region of the pharynx (arrow); (X) prominent Sgg-PO expression in the proventriculus (arrow) and (Y) Sgg-PP in the hindgut (arrow). Scale bar in V = 20μm applies to V-Y. (PDF) [file pone.0236679.s002.pdf]
